# Massive Shift in Gene Expression during Transitions between Developmental Stages of the Gall Midge, *Mayetiola Destructor*

**DOI:** 10.1371/journal.pone.0155616

**Published:** 2016-05-25

**Authors:** Ming-Shun Chen, Sanzhen Liu, Haiyan Wang, Xiaoyan Cheng, Mustapha El Bouhssini, R. Jeff Whitworth

**Affiliations:** 1 Hard Winter Wheat Genetics Research Unit, USDA-ARS, Kansas State University, Manhattan, Kansas, United States of America; 2 Department of Entomology, Kansas State University, Manhattan, Kansas, United States of America; 3 Department of Plant Pathology, Kansas State University, Manhattan, Kansas, United States of America; 4 Department of Statistics, Kansas State University, Manhattan, Kansas, United States of America; 5 International Center for Agricultural Research in the Dry Area, Rabat, Morocco; Laboratoire Arago, FRANCE

## Abstract

*Mayetiola destructor* is a destructive pest of wheat and has six developmental stages. Molecular mechanisms controlling the transition between developmental stages remain unknown. Here we analyzed genes that were expressed differentially between two successive developmental stages, including larvae at 1, 3, 5, and 7 days, pupae, and adults. A total of 17,344 genes were expressed during one or more of these studied stages. Among the expressed genes, 38–68% were differently expressed between two successive stages, with roughly equal percentages of up- and down-regulated genes. Analysis of the functions of the differentially expressed genes revealed that each developmental stage had some unique types of expressed genes that are characteristic of the physiology at that stage. This is the first genome-wide analysis of genes differentially expressed in different stages in a gall midge. The large dataset of up- and down-regulated genes in each stage of the insect shall be very useful for future research to elucidate mechanisms regulating insect development and other biological processes.

## Introduction

Gall midges are flies of the family Cecidomyiidae, one of the largest families within the order of Diptera [1, 2]. Many gall midge species possess fascinating biological traits such as extraordinary ability to manipulate host plants [3], fast adaptation to host defenses (resistance) [4, 5], genomic imprinting, and extensive E-chromosome elimination [6–8]. A great portion of gall midge species are also economically important and cause significant damage to crops. A few examples of economically important gall midge species include the Asian rice gall midge *Orseolia oryzae* [3], the orange wheat blossom midge *Sitodiplosis mosellana* [9], the barley stem midge *Mayetiola hordei* [10], the saddle gall midge *Haplodiplosis margina* [11], the blueberry gall midge *Dasineura oxycoccana* [12], the honey locust pod gall midge *Dasineura gleditschiae* [13], and the citrus gall midge *Prodiplosis longifila* [14]. Despite their fascinating biology and great economic importance, the gall midges as a group are molecularly and genomically under-studied.

*Mayetiola destructor*, commonly known as the Hessian fly, is a member of the Cecidomyiidae family and a global destructive pest of wheat [8]. Because of its economic importance as an agricultural pest, the Hessian fly has been the most extensively studied pest at the molecular level, and is the only gall midge species whose genome has been sequenced [15]. Like other gall midge species, the Hessian fly has unique biological and genetic traits, including irreversibly manipulating host plants [16, 17], rapid biotype sweeps [18, 19], dramatic expansion and unconventional conservation of effector-type genes [15, 20], and dramatic expansion of microRNA-encoding genes [21]. The Hessian fly pest is currently controlled by three main approaches: 1) planting wheat late to avoid early infestation; 2) destroying volunteer wheat plants; and 3) deployment of resistant cultivars [3, 8]. Each tactic can be successful under certain conditions, but none of them can prevent Hessian fly damage, and wheat loss due to Hessian fly can still reach 5% of gross production annually [22].

One of the high throughput methods for global analysis of gene expression is the technology called RNA sequencing (RNA-Seq) [23, 24]. RNA-Seq has been used to analyze changes in gene expression at different stages of development or the same developmental stage under different environments of many insect species, including *Drosophila melanogaster* [25], *Aedes albopictus* [26, 27], *A*. *aegypti* [28], *Calanus finmarchicus* [29], *Adelphocoris suturalis* [30], *Coleomegilla maculata* [31], and *Meligethes aeneus* [32]. Like the highly studied *D*. *melanogaster*, Hessian fly has six different developmental stages including eggs, three instars of larvae, pupae, and adults; and each stage of Hessian fly has unique biology [7, 8]. Hessian fly larvae, the only feeding stage, live within wheat plants as parasites. The 1^st^ instar larvae (lasting for four days at 20°C) is the critical stage to manipulate host plants such as inhibiting wheat growth and inducing the formation of nutritive cells. Failure to manipulate host plants will result in the death of Hessian fly larvae. Early age of 1^st^ instar (fresh to 1-day larvae) is most active in secreting effectors for plant manipulation, whereas later age of 1^st^ instar (3 to 4-day larvae) is most active in secreting proteins that counteract plant defense [16, 33]. Hessian fly larvae at 1^st^ instar produce a large number of secreted salivary gland proteins (SSGPs). The specific functions of SSGPs in Hessian fly are not known, but are presumably injected into host plants, serving as effectors during plant manipulation [15, 20, 34, 35]. Hessian fly larvae transform from 1^st^ into 2^nd^ instar at day 5, and transform from 2^nd^ into 3^rd^ instar at day 10 under our experimental conditions. The objective of this study is to determine changes in gene expression between different ages of first instar larvae and during transitions between two successive developmental stages of Hessian fly via RNA-Seq. Such information should provide a foundation for further analysis to identify molecular mechanisms for the unique biological and genetic traits of Hessian fly, and to identify molecular targets that are suitable for developing more effective measures to control Hessian fly damage.

## Results

To understand the change in gene expression at different developmental stages, samples from multiple developmental stages, including larvae at 1, 3, 5, and 7 days, pupae, and adults, were subjected to mRNA sequencing (RNA-Seq) using the Illumina HiSeq2000 sequencing platform, of which three biological replicates were conducted. Read counts between the replicates are highly correlated (>97% on average), indicating the repeatability is quite high. On average 33.5 million of 2x101 bp paired-end raw reads per sample, ranging from 26.2–41.4 millions, were obtained. The raw reads were subjected to adaptor and quality trimming using Trimmomatic [36]. On average, 95% clean reads were obtained after quality trimming and ~96.7% clean reads can be mapped to the reference genome, indicating that the sequence quality was good and contamination was low if any. The clean reads were then mapped to the Hessian fly draft genome sequence (Mdes20100623) via GSNAP, an intron-aware aligner [37]. As a result, on average 96.7% clean reads were mapped and 88.1% were uniquely mapped. These uniquely mapped reads were used for further read counting per gene for gene expression analyses.

### Dramatic changes in up- and down-regulated genes between different developmental stages

A draft Hessian fly genome sequence and predicted gene models were used as reference sequences (https://i5k.nal.usda.gov/Mayetiola_destructor) [15]. A total of 17,344 genes were found to have the corresponding reads detected in the RNA-Seq samples from at least one of the developmental stages (S1 Table). The abundance of the reads per gene was then compared between two successive stages of the insect using an R package DESeq2 [38], namely, 3- versus 1-day larvae, 5- versus 3-day larvae, 7- versus 5-day larvae, pupae versus 7-day larvae, and adults versus pupae (Table 1 and S2 Table). Informative genes, genes remained after filtering those unlikely to be differentially expressed due to low read counts by using the default DESeq2 setting, in the comparison between these samples ranged from 16,275 to 17,133. Using the threshold of 5% false discovery rate, 8,479 (49.5%), 7,265 (44.4%), and 6,249 (38.4%) genes exhibited significant differences between samples from different larval stages, whereas 11,539 (67.7) and 10,414 (63.9) genes exhibited significant differences between samples from pupae and 7-day larvae, and between samples from adults and pupae. The total number of genes with significant difference in transcript levels was roughly equally (˂2% differences) distributed between up- and down-regulated genes among comparisons between two larval stages (Table 1, S1 and S2 Figs). However, there were about 7% more genes down-regulated than up-regulated between the pupal and 7-day larval samples, and about 5% more genes up-regulated than down-regulated between the adult and pupal samples. In addition, the magnitudes of up- and down-regulation varied among different comparisons. For example, there were approximately twice as many genes up-regulated four or more times than those down-regulated between 3- versus 1-day larvae, 5- versus 3-day larvae, and adult versus pupae. In contrast, there were more than twice as many genes down-regulated four or more times compared with those up-regulated between pupal versus 7-day old larvae.

**Table 1:** Total number (No) of informative (Inf.) genes, number of genes with significant (Sign.) change in expression when samples from two successive stages of Hessian fly were compared, and fold change of up- or down-regulation. L, P, and A represent larvae, pupae and adults.

Comparison	Inf. genes	Sign. genes	Up-regulation				Down-regulation			
			Total	1–2 fold	2–4 fold	≥4 fold	Total	1–2 fold	2–4 fold	≥4 fold
3- vs 1-day L No	17133	8479	4388	2416	767	1205	4091	2097	1471	523
%	100	49.5	25.6	14.1	4.5	7.0	23.9	12.2	8.6	3.1
5- vs 3-day L No	16351	7265	3721	1300	1074	1347	3544	2071	781	692
%	100	44.4	22.8	8.0	6.7	8.2	21.6	12.7	4.8	4.2
7- vs 5-day L No	16275	6249	3148	2307	662	179	3101	1562	1222	317
%	100	38.4	19.3	14.2	4.1	1.1	19.1	9.6	7.5	1.9
P vs 7-day L No	17053	11539	5188	2123	1807	1258	6351	1848	1522	2981
%	100	67.7	30.4	12.4	10.6	7.4	37.2	10.8	8.9	17.5
A vs P No	16292	10414	5536	712	1345	3479	4878	1237	1974	1667
%	100	63.9	34.0	4.4	8.3	21.4	29.9	7.6	12.1	10.2

### Classification of expressed genes

To examine what type of genes were up- or down-regulated between samples from two successive developmental stages, the 17,344 informative genes were compared with sequences in Genbank using BLASTX. The BLASTX analysis revealed that 9,162 (52.8%) of the informative genes had matches with Genbank sequences with E-values equal or smaller than 1e^-30^. Among the 9,162 genes with Genbank matches, 5,554 (32.0% of the total informative genes) were with known functions according to the annotations in the database of the Universal Protein Resources (UniProt) [39] (http://www.uniprot.org/uniprot/). The 5,554 genes with functions known, or so called known genes, were divided into eight different functional categories according to their Gene Ontology (GO) terms [40] (Table 2 and S3 Table). The eight functional categories include ‘nutrient (general) metabolism’ (811 or 14.6%), ‘reduction/oxidation (redox)/detoxification’ (97 or 1.7%), ‘structure and adhesion’ (294 or 5.3%), ‘RNA metabolism’ (363 or 6.5%), ‘protein metabolism’ (835 or 15.1%), ‘transport’ (777 or 14.0%), ‘regulatory proteins’ (2,002 or 36.0%) and ‘secreted salivary gland proteins (SSGPs)’ (376 or 6.8%). Proteins encoded by SSGP genes in the Hessian fly genome are unique with no meaningful sequence similarity to known proteins in public databases [20]. SSGPs are likely injected into host tissues during feeding to manipulate host physiology, but specific functions for individual SSGPs are not known. Genes in each category were further classified into subcategories as shown in Table 2.

**Table 2:** Functional categories (bold) and subcategories of genes with known functions, and percentages of genes in each category and subcategory that were up- (U) or down-regulated (D) between two successive larval stages. TD (Times of differences) represents the magnitude of difference between up- and down-regulated genes, and was calculated by dividing the bigger percentage with the smaller percentage in the same comparison. The symbol '+' indicates more up-regulated genes whereas the symbol '-' indicates more down-regulated genes.

Functional category and subcategory	No. of genes (%)	3- vs 1-day larvae			5- vs 3-day larvae			7- vs 5-day larvae		
		% D	% U	TD	% D	% U	TD	% D	% U	TD
**Nutrient metabolism**	**811 (14.6)**	**13.4**	**34.2**	**+2.6**	**5**	**44.6**	**+8.9**	**7.5**	**41.5**	**+5.5**
Carbohydrate	145 (17.9)	7.6	33.1	+4.4	4.8	48.3	+10.1	8.3	41.4	+5.0
TCA cycle/energy	117 (14.3)	5.1	48.7	+9.5	1.7	47	+27.6	1.7	56.4	+33.2
Lipid	171 (21.1)	18.6	25	+1.3	6.4	52.3	+8.2	14	33.1	+2.4
Amino acid	203 (25.0)	12.3	36.8	+3	2	39.2	+19.6	3.9	41.7	+10.7
Others	176 (21.7)	19.9	31.3	+1.6	9.7	38.6	+4.0	8.5	39.8	+4.7
**Redox/detoxification**	**97 (1.7)**	**24.7**	**38.1**	**+1.5**	**6.2**	**36.1**	**+5.8**	**12.4**	**39.2**	**+3.2**
Cytochrome P450s	56 (57.7)	39.3	25	-1.6	8.9	33.9	+3.8	14.3	42.9	+3.0
Glutathione transferases	12 (12.4)	16.7	58.3	+3.5	8.3	41.7	+5.0	8.3	25	+3.0
Peroxidases	11 (11.3)	0	54.5	+++	0	45.5	+++	27.3	27.3	1
Others	18 (18.6)	0	55.6	+++	0	33.3	+++	0	44.4	+++
**Structure and adhesion**	**294 (5.3)**	**19.4**	**31**	**+1.6**	**10.2**	**32**	**+3.1**	**25.5**	**23.1**	**-1.1**
Structural	151 (51.4)	23.2	32.5	+1.4	6	33.8	+5.6	16.6	34.4	+2.1
Adhesion	108 (36.7)	18.5	29.6	+1.6	18.5	29.6	+1.6	38.9	10.2	-3.8
Cuticle proteins	13 (4.4)	0	30.8	+++	0	53.8	+++	30.8	7.7	-4.0
Others	22 (7.5)	9.1	27.3	+3	4.5	18.2	+4.0	18.2	18.2	1
**RNA metabolism**	**363 (6.3)**	**8**	**28.7**	**+3.6**	**14.3**	**8.8**	**-1.6**	**9.9**	**9.9**	**1**
RNA helicases	42 (11.6)	14.3	26.2	+1.8	14.3	0	---	14.3	4.8	-3
RNA modification	41 (11.3)	2.4	22	+9.2	7.3	2.4	-3.0	0	7.3	+++
RNA processing	164 (45.2)	7.9	24.4	+3.1	18.9	11.6	-1.6	14.6	7.9	-1.9
Ribonucleases	28 (7.7)	14.3	17.9	+1.3	10.7	17.9	+1.7	0	17.9	+++
tRNA synthesis	48 (13.2)	2.1	60.4	+28.8	8.3	12.5	+1.5	0	22.9	+++
RNA degradation	17 (4.7)	5.9	41.2	+7.0	5.9	5.9	+1.0	11.8	0	---
Others	23 (6.3)	13	13	+1.0	17.4	0	---	17.4	8.7	-2.0
**Protein metabolism**	**835 (15.0)**	**15.1**	**36.8**	**2.4**	**7.4**	**20.2**	**+2.7**	**7.8**	**30.8**	**+3.9**
Ribosomal proteins	95 (11.4)	1.1	80	+72.7	2.1	11.6	+5.5	0	52.6	+++
Protein translation	55 (6.6)	3.6	52.7	+14.6	3.6	7.3	+2.0	7.3	18.2	+2.5
Folding/chaperones	139 (16.6)	9.4	42.4	+4.5	9.4	8.6	-1.1	2.2	24.5	+11.1
Protein modification	124 (14.9)	14.5	27.4	+1.9	4	23.4	+5.9	5.6	36.3	+6.5
Proteasome/ubiquilation	221 (26.5)	25.3	26.2	+1.0	6.8	16.7	+2.5	6.8	28.5	+4.2
Proteases	173 (20.6)	17.3	23.7	+1.4	12.7	35.3	+2.8	17.3	26	+1.5
Protease inhibitor	30 (3.5)	20	36.7	+1.8	10	50	+5.0	20	36.7	+1.8
**Transport**	**777 (14.0)**	**20.7**	**25.4**	**+1.2**	**12.6**	**25.3**	**+2.0**	**15**	**29.4**	**+2.0**
Retrograde	21 (2.7)	19.1	19	1	9.5	28.6	+3.0	14.3	28.6	+2.0
Amino acid	32 (4.1)	18.8	12.5	-1.5	12.5	46.9	+3.8	9.4	34.4	+3.7
Ion	188 (24.2)	30.3	20.7	-1.5	19.2	26.6	+1.4	21.8	29.3	+1.3
Sugar	24 (3.1)	16.7	12.5	-1.3	16.7	41.7	+2.5	16.7	45.8	+2.7
Lipid/fatty acid	36 (4.6)	22.2	33.3	+1.5	11.1	52.8	+4.8	13.9	36.1	+2.6
Protein	187 (24.1)	12.3	31	+2.5	4.8	16.6	+3.5	9.6	34.8	+3.6
RNA	28 (3.6)	7.1	42.9	+6.0	14.3	3.6	-4.0	14.3	7.1	-2.0
Neuro-transmitter	36 (4.6)	22.2	8.3	-2.7	16.7	22.2	+1.3	19.4	19.4	1
Others	226 (29.0)	21.7	27.9	+1.3	12.8	25.2	+2.0	14.2	26.1	+1.8
**Regulatory proteins**	**2002 (36.0)**	**24.6**	**19**	**-1.3**	**29.4**	**16.5**	**-1.8**	**18.8**	**15.2**	**-1.2**
Apoptosis	35 (1.7)	22.9	28.6	+1.2	8.6	28.6	+29.3	11.4	34.3	+3.0
Cell cycle	145 (7.2)	22.1	30.3	+1.4	15.9	10.3	-1.5	25.5	14.5	-1.8
Chromatin	106 (5.3)	20.8	15.1	-1.4	10.4	7.5	-1.4	7.5	20.8	+2.8
Growth/development	269 (13.4)	22.3	19.3	-1.2	17.1	19.3	+1.1	24.5	14.5	-1.7
Helicases/DNA repair	65 (3.2)	12.3	29.2	+2.4	20	4.6	-4.3	7.7	10.8	+1.4
DNA replication	36 (1.8)	5.6	77.8	+13.9	8.3	5.6	-1.5	30.6	5.6	-5.5
Gene silencing	23 (1.1)	13	39.1	+3.0	8.7	26.1	+3.0	21.7	43.5	+2.0
Immunity/defense	38 (1.9)	18.4	26.3	+1.4	7.9	23.7	+3.0	13.2	42.1	+3.2
Nucleases	45 (2.2)	8.9	20	+2.2	2.2	15.6	+7.1	4.4	20	+4.5
Sensory transduction	49 (2.4)	36.7	10.2	-3.6	26.5	12.2	-2.2	20.4	8.2	-2.5
Signal transduction	621 (31.0)	30.4	16.4	-1.9	14.5	21.9	+1.5	20.8	17.1	-1.2
Transcription	527 (26.4)	26.4	14.2	-1.9	16.7	13.5	-1.2	17.8	9.9	-1.8
Others	42 (2.1)	11.9	2.4	-5	4.8	14.3	+3.0	2.4	9.5	+4.0
**SSGPs**	**376 (6.8)**	**8.8**	**84**	**+9.5**	**61.4**	**10.9**	**-5.6**	**38.8**	**5.1**	**-7.7**

### Unbalanced changes between up- and down-regulation among functional gene-categories

**Fig 1:**
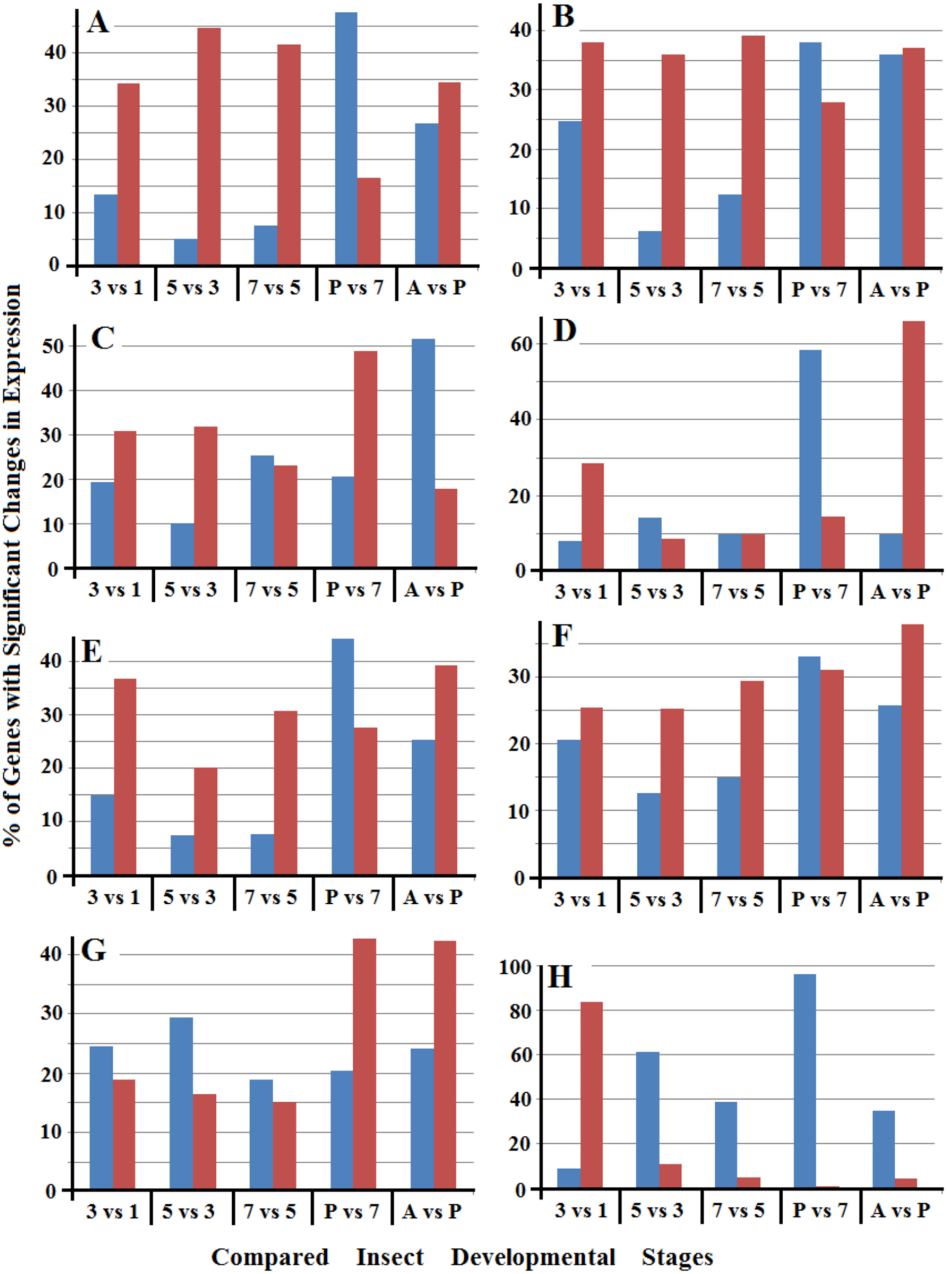
Percentages of up- (red bars) and down-regulated (blue bars) genes between two successive stages of Hessian fly in eight different functional categories. ‘3 vs 1’, ‘5 vs 3’, ‘7 vs5’, ‘P vs 7’, and ‘A vs P’ represent comparisons made between 3- versus 1-day larvae, 5- versus 3-day larvae, 7- versus 5-day larvae, pupae versus 7-day larvae, and adults versus pupae. The eight categories of genes were indicated on the top of each graph in the figure.

Even though the total percentages of up- and down-regulated genes were roughly the same between samples from two successive developmental stages of the insect, further analysis of different categories of the genes with altered expression revealed dramatic differences among different gene types. As shown in Fig 1, the percentages of up-regulated genes were quite different from those of down-regulated genes in each category with only a couple of exceptions. For example, 34–48% of the ‘nutrient metabolism’ genes were up-regulated between samples from two successive larval stages, but only 5–14% of the ‘nutrient metabolism’ genes were down-regulated between these samples (Fig 1A). In contrast, over 50% of the ‘nutrient metabolism’ genes were down-regulated in pupae when it was compared with 7-day old larvae, yet only ~15% of the ‘nutrient metabolism’ genes were up-regulated when these two samples were compared. Another example is the dramatic difference between up- and down-regulated genes in the category ‘SSGPs’. More than 80% of SSGP-encoding genes were up-regulated when 3-day old larvae were compared with 1-day old larvae. In contrast, less than 9% of the SSGP genes were down-regulated when these two samples were compared. Interestingly, ~60% of SSGP genes were down-regulated when 5-day old larvae were compared with 3-day old larvae, whereas less than 11% of the SSGP genes were up-regulated between these samples. Higher percentages of SSGP genes were further down-regulated as the insect aged into later developmental stages (Fig 1H). Unbalanced changes between up- and down-regulated genes were also observed in most other categories of genes when samples from two successive developmental stages of Hessian fly were compared (Fig 1B to 1G).

### Genes with altered expression between two larval stages

The genes with altered expression in each category were further classified into subcategories as described earlier. For ‘nutrient metabolism’, much greater percentages of genes in all subcategories were up-regulated than down-regulated between 3- versus 1-day old larvae, 5- versus 3-day old larvae, or 7- versus 5-day old larvae. Particularly for ‘TCA cycle’ and ‘amino acid metabolism’, up-regulated genes outnumbered down-regulated as much as 30 times. A similar situation was also observed in ‘redox/detoxification’, in which more genes were up-regulated than down-regulated in all subcategories, except for ‘cytochrome P450s’ between 3- versus 1-day larvae and ‘peroxidases’ between 7- versus 5-day old larvae.

For ‘structure and adhesion’, the situation was somehow different. For the subcategory ‘structural components’, higher percentages of up-regulated genes were observed among all comparisons between two larval stages. However, for the subcategories ‘adhesion molecules’ and ‘cuticle proteins’, higher percentages of up-regulated genes were only observed in comparisons between 3- versus 1-day larvae and 5- versus 3-day larvae. In contrast, down-regulated genes outnumbered up-regulated genes more than three times in the comparison between 7- versus 5-day larvae.

For ‘RNA metabolism’, genes in each subcategory behaved very differently in different comparisons. Between 3- versus 1-day larvae, higher percentages of up-regulated genes were observed in all subcategories except for ‘others’. Particularly for genes in ‘RNA modification’, ‘tRNA synthesis’, and ‘RNA degradation’, up-regulated genes outnumbered down-regulated genes 7–29 times between 3- versus 1-day larvae. However, the situations were different and five of the seven subcategories had equal or more genes down-regulated between 5- versus 3-day larvae. The situation changed again in the comparison between 7- versus 5-day larvae, when four subcategories of genes had higher percentages of down-regulated genes and three sub-categories of genes had higher percentages of up-regulated genes. For ‘protein metabolism’, more genes were up-regulated in all subcategories among all comparisons except for ‘protein folding/chaperones’ between 5- versus 3-day larvae. It is particularly interesting to note that all ribosomal protein genes except for one were up-regulated when larvae aged from 1-day to 3-days.

For ‘transport’, higher percentages of down-regulated genes were found in the subcategories ‘amino acid transport’, ‘ion transport’, ‘carbohydrate transport’, and ‘neuro-transmitter transport’ between 3- versus 1-day larvae; whereas higher percentages of up-regulated genes were observed in the subcategories ‘lipid/fatty acid transport’, ‘protein transport’, ‘RNA transport’, and ‘others’ between these samples. However, higher percentages of up-regulated genes were observed in all subcategories except for ‘RNA transport’ when 5- versus 3-day larvae were compared. Higher percentages of up-regulated genes were observed in all subcategories except for ‘RNA transport’ and ‘neuro-transmitter transport’ when 7- versus 5-day larvae were compared. For ‘regulatory proteins’, genes in each subcategory showed a unique pattern among the three comparisons between different larval stages. For the subcategories ‘apoptosis’, ‘gene silencing’, ‘immunity/defense’, and ‘nucleases’, higher percentages of up-regulated genes were observed among all three larval stage comparisons. In contrast, for ‘sensory transduction’ and ‘transcription’, higher percentages of down-regulated genes were found among all three larval comparisons. For genes in other subcategories, inconsistent patterns were observed among these three larval comparisons.

For ‘SSGPs’, 84% genes were up-regulated and only 8.8% genes were down-regulated when comparison was made between 3- versus 1-day larvae. On the other hand, much higher percentages of down-regulated ‘SSGP’ genes were observed when comparisons were made between 5- versus 3-day old larvae, and between 7- versus 5-day larvae.

### Genes with altered expression between two stages of morphogenesis

The transition from larvae to pupae and from pupae to adults is defined as different stages of morphogenesis. The changes of gene expression between two stages of morphogenesis were remarkably different from those between two larval growth stages. For the genes in the category ‘nutrient metabolism’, much greater percentages (1.8 to 18.5 fold) of genes were down-regulated than those up-regulated in all subcategories between pupae versus 7-day old larvae (Table 3). Particularly, 63.8% genes in the subcategory ‘TCA cycle/energy’ were down-regulated, whereas only 3.4% of the genes in this subcategory were up-regulated. This observation was opposite of what was observed in the comparisons between two larval stages. Activity of nutrient metabolism recovered somewhat in adults since there were slightly more up-regulated than down-regulated genes in all subcategories, except for the subcategory ‘carbohydrate metabolism’, when the samples from adults were compared with the samples from pupae.

**Table 3:** Percentages of genes in each category (bold) and subcategory that were up- (U) or down-regulated (D) between samples from two successive stages of morphogenesis. TD (Times of differences) represents the magnitude of difference between up- and down-regulated genes, and was calculated by dividing the bigger percentage with the smaller percentage in the same comparison. The symbol '+' indicates more up-regulated genes whereas the symbol '-' indicates more down-regulated genes.

Functional category and subcategory	No. of genes	Pupae vs 7-day larvae			Adults vs pupae		
		% D	% U	TD	% D	% U	TD
**Nutrient metabolism**	**811**	**47.7**	**16.4**	**-2.9**	**26.8**	**34.6**	**+1.3**
Carbohydrate	145	42.8	14.5	-3.0	29.6	24.1	-1.2
TCA cycle/energy	116	63.8	3.4	-18.5	16.4	31	+1.9
Lipid	171	40.4	22.8	-1.8	33.3	36.3	+1.1
Amino acid	203	52.7	16.3	-3.2	24.6	41.4	+1.7
Others	176	42.6	20.5	-2.1	27.3	36.4	+1.3
**Redox/detoxification**	**97**	**38.1**	**27.8**	**-1.4**	**36.1**	**37.1**	**-1.0**
Cytochrome P450s	56	33.9	37.5	+1.1	46.4	35.7	-1.3
Glutathione transferases	12	33.3	25	-1.3	16.7	50	+3.0
Peroxidases	11	54.5	9.1	-6.0	36.4	18.2	-2.0
Others	18	44.4	5.6	-7.9	16.7	44.4	+2.7
**Structure and adhesion**	**294**	**20.7**	**48.6**	**+2.3**	**51.7**	**18**	**-2.9**
Structural	151	25.8	43	+1.7	45.7	24.5	-1.9
Adhesion	108	12	62	+5.2	58.3	12	-4.8
Cuticle proteins	13	15.4	23.1	+1.7	61.5	7.7	-8.0
Others	22	31.8	36.4	+1.1	54.5	9.2	-6.0
**RNA metabolism**	**363**	**58.4**	**14.6**	**-4.0**	**9.9**	**65.8**	**+6.6**
RNA helicases	42	50	23.8	-2.1	9.5	64.3	+6.8
RNA modification	41	62	9.8	-6.3	2.4	68.3	+28.5
RNA processing	164	53.7	18.9	-2.8	12.8	65.9	+5.1
Ribonucleases	28	57.1	10.7	-5.3	14.3	71.4	+5.0
tRNA synthesis	48	81.3	0	---	6.3	54.2	+8.6
RNA degradation	17	58.8	23.5	-2.5	11.8	70.6	+6.0
Others	23	56.5	4.3	-13.0	4.4	78.3	+17.8
**Protein metabolism**	**837**	**44.4**	**27.5**	**-1.6**	**25.5**	**39.5**	**+1.5**
Ribosomal proteins	95	92.6	1.1	-88.0	34.7	42.1	+1.2
Protein translation	55	67.3	9.1	-7.4	21.8	41.8	+1.9
Folding/chaperones	139	36.7	27.3	-1.3	23.7	32.4	+1.4
Protein modification	124	43.5	25	-1.7	24.2	43.5	+1.8
Proteasome/ubiquilation	221	29.4	43.4	+1.5	19	43.4	+2.3
Proteases	173	36.6	28.5	-1.3	28.5	37.8	+1.3
Protease inhibitor	30	44.8	34.5	-1.3	51.7	24.1	-2.1
**Transport**	**778**	**33.2**	**30.9**	**-1.1**	**25.9**	**38**	**+1.5**
Retrograde	21	33.3	23.8	-1.4	19	47.6	+2.5
Amino acid	32	28.1	34.3	+1.2	25	43.8	+1.8
Ion	188	34.6	27.1	-1.3	33.5	28.7	-1.2
Sugar	24	62.5	20.8	-3.0	16.7	50	+3.0
Lipid/fatty acid	36	19.4	33.3	+1.7	41.7	25	-1.7
Protein	187	36.9	32.6	-1.1	21.4	39	+1.8
RNA	28	35.7	32.1	-1.1	7.1	60.7	+8.5
Neuro-transmitter	36	13.9	30.6	+2.2	25	47.2	+1.9
Others	226	31.6	33.3	+1.1	24.9	39.6	+1.6
**Regulatory proteins**	**2001**	**20.5**	**42.7**	**+2.1**	**24.2**	**42.4**	**+1.8**
Apoptosis	35	20	48.6	+2.4	40	48.6	+1.2
Cell cycle	145	25.5	32.4	+1.3	7.6	66.9	+8.8
Chromatin	106	21.7	27.4	+1.3	14.2	58.5	+4.1
Growth/development	269	15.6	56.1	+3.6	36.8	34.2	-1.1
Helicases/DNA repair	65	30.8	33.8	+1.1	9.2	72.3	+7.9
DNA replication	36	61.1	13.9	-4.4	5.6	88.9	+15.9
Gene silencing	23	17.4	60.9	+3.5	30.4	21.7	-1.4
Immunity/defense	38	31.6	36.8	+1.2	44.7	28.9	-1.5
Nucleases	45	40	13.3	-3.0	17.8	31.1	+1.7
Sensory transduction	49	14.3	32.7	+2.3	24.5	38.8	+1.6
Signal transduction	621	18.5	49	+2.6	25.8	37.7	+1.5
Transcription	527	17.8	42.2	+2.4	25.4	40.5	+1.6
Others	42	21.4	16.7	-1.3	16.7	9.52	-1.6
**SSGPs**	**376**	**96.8**	**0.8**	**-121**	**34.9**	**4.3**	**-8.2**

For the genes in the category ‘redox/detoxification’, again more genes were down-regulated than up-regulated, except for the subcategory ‘P450s’, which had slightly less genes down-regulated than up-regulated, when 7-day old larvae were compared with pupae. During the transition from pupae to adults, more genes in the subcategories ‘P450s’ and ‘peroxidases’ were down-regulated than up-regulated. However, more genes were up-regulated in the subcategories ‘glutathione transferases’ and ‘others’.

For the genes in the category ‘structure and adhesion’, higher percentages of genes were up-regulated than down-regulated in all subcategories when pupae were compared with 7-day old larvae. The opposite occurred, namely higher percentages of genes were down-regulated in all subcategories, when adults were compared with pupae.

For the genes in the category ‘RNA metabolism’, at least twice as many genes were down-regulated than up-regulated in all subcategories and at least 50% of the genes were down-regulated in each subcategory between samples from pupae versus samples from 7-day old larvae. In contrast, at least five times more genes were up-regulated than down-regulated in all subcategories, and at least 54% of genes were up-regulated in each subcategory when adults were compared with pupae.

Genes in the category ‘protein metabolism’ had higher percentages of genes down-regulated versus up-regulated in all subcategories between pupae and 7-day old larvae, except for the subcategory ‘proteasome/ubiquilation’, which had a slightly higher percentage of up-regulated genes between these two samples. Over 92% of the genes in the subcategory ‘ribosomal proteins’, and over 67% of the genes in the subcategory ‘protein translation’ were down-regulated during the larva-pupa transition, indicating protein synthesis was highly suppressed in pupae. Conversely, higher percentages of genes were up-regulated when samples from adults were compared with samples from pupae in all subcategories, except for the subcategory ‘protease inhibitor’, which had 2.1 times more down-regulated genes.

Genes in the category ‘transport’ had slightly higher percentages of genes down-regulated in the subcategories ‘retrograde transport’, ‘ion transport’, ‘protein transport’, and ‘RNA transport’; and threefold more genes down-regulated versus up-regulated in the subcategory ‘sugar transport’ when the samples from pupae were compared with samples from 7-day old larvae. In contrast, a slightly higher percentage of genes were up-regulated for the subcategories ‘amino acid transport’ and ‘others’. Also a moderately higher percentage of genes was up-regulated than down-regulated in the subcategories ‘lipid/fatty acid transport’, and more than twice as many genes were up-regulated in the subcategory ‘neuro-transmitter transport’ during the larva-pupa transition. In comparison, higher percentages of genes were up-regulated than down-regulated in all subcategories except for ‘ion transport’ and ‘lipid/fatty acid transport’, which had slightly higher percentages of genes down-regulated when samples from adults were compared with samples from pupae.

For genes in the category ‘regulatory proteins’, higher percentages of genes were up-regulated in the subcategories ‘apoptosis’, ‘cell cycle’, ‘chromatin’, ‘growth/development’, ‘helicases/DNA repair’, ‘gene silencing’, ‘immunity/defense’, ‘sensory transduction’, ‘signal transduction’, and ‘transcription’; but higher percentages of genes were down-regulated in the subcategories ‘DNA replication’, ‘nucleases’, and ‘others’ when samples from pupae were compared with samples from 7-day larvae. During the transition of the insect from pupa to adult, higher percentages of genes were up-regulated than down-regulated in all subcategories except for ‘growth/development’, in which similar numbers of genes were either up-regulated or down-regulated; and the subcategories ‘gene silencing’, ‘immunity/defense’, and ‘others’, in which slightly higher percentages of genes were down-regulated.

Over 96% of genes encoding SSGPs were down-regulated during the transition of the insect from 7-day old larvae to pupae. During the transition of the insect from pupae to adults, 34.9% SSGP-encoding genes were further down-regulated compared with only 4.3% SSGP-encoding genes up-regulated.

## Discussion

Genome-wide analysis of gene expression generates large datasets, and because of that, most studies report results with whole datasets analyzed through various standardized methods such as GO categorization, Clusters of Orthologous Groups (COG), and Kyoto Encyclopedia of Genes and Genomes (KEGG) classification [25–32]. These types of analysis do provide very useful information on the overall pictures of changes in gene expression. However, these types of reports often do not provide readers much information on changes linked to specific biochemical pathways, for example, glycolysis or protein synthesis. In this study, we divided the whole sets of informative genes detected in Hessian fly into four groups: genes with no BLASTx match in Genbank, genes with matches to functionally unknown sequences, genes with matches to sequences with known functions, and genes encoding SSGPs. We then further classified the genes with known functions into different categories and subcategories according to their specific functions [39, 40]. Based on these functional classifications, we compared differences in gene expression of Hessian fly between two successive larval growth stages and stages of morphogenesis from larva to pupa and from pupa to adult. We found that the expression levels of 38–50% of genes were significantly altered between two successive larval stages, and that the expression levels of 63–68% genes were significantly changed between two stages of morphogenesis. In each comparison, the overall percentages of up-regulated genes were similar to those of down-regulated genes, even though the magnitudes of up- and down-regulation were different from comparison to comparison. However, when the genes in each functional category or subcategory were analyzed separately (based on the same whole dataset statistics), the percentages of up-regulated genes were remarkably different from the percentages of down-regulated genes among different gene categories or subcategories and between different developmental stages of the insect. This observation indicated that different physiological and biochemical pathways were shifted up or turned down during different developmental stages.

### Changes in gene expression and pathways during larval development

One of the remarkable common alterations in gene expression during larval growth stages was that up-regulated genes dramatically outnumbered down-regulated genes in the category ‘nutrient metabolism’ (Table 2). As much as 10 times more metabolic genes were up-regulated versus down-regulated when comparisons were made between two successive larval stages. The up-regulation of ‘nutrient metabolism’ genes was across all different subcategories. Among them, up-regulated genes in ‘TCA cycle/energy metabolism’ outnumbered down-regulated genes the most, suggesting that nutrient metabolism increased steadily during all larval stages to provide energy and intermediates for larval growth and development.

Genes in categories other than ‘nutrient metabolism’ were regulated differently among different larval growth stages. During the 1–3 day larval stage, the up-regulation of nutrient metabolism pathways was apparently to enhance the overall activity of transcription and translation. Up-regulated genes in the category ‘RNA metabolism’ outnumbered down-regulated genes in all subcategories except for ‘others’, indicating that activity of RNA transcription and processing was enhanced during the 1–3 day larval period. The genes in the subcategory ‘tRNA synthesis’ outnumbered hugely down-regulated genes. The ‘tRNA synthesis’ genes included various tRNA synthetases or tRNA ligases, which are required for protein translation. The broad up-regulation of tRNA synthetase or ligase genes indicated that protein synthesis increased during this early larval stage. Consistent with this notion, up-regulated genes in the subcategories ‘ribosomal proteins’, ‘protein translation’ and ‘protein folding/chaperones’ also hugely outnumbered down-regulated genes. Ribosomal proteins, translation initiation factors, translation elongation factors, and chaperones for protein folding all participate directly in protein synthesis. The enhancement of transcription and translation activities during the 1–3 day larval period was further supported by the greater percentages of up-regulated than down-regulated genes of those involved in RNA and protein transport. The enhancement of the overall transcription and translation activity in the 1–3 day larval stage might provide the molecular basis for rapid larval growth [16].

It is essential for first instar larvae to suppress host defense, inhibit plant growth, and establish a permanent feeding site. Failure to achieve these will result in larval death [7, 8]. To manipulate host plants successfully, Hessian fly larvae inject effector proteins, namely various SSGPs, into host tissues [15, 20]. Genes encoding SSGPs were up-regulated broadly and specifically during the 1–3 larval period. In addition, up-regulated genes also outnumbered down-regulated genes in the categories ‘redox/detoxification’. Specifically, genes encoding glutathione transferases, peroxidases, superoxide dismutases, and catalases were up-regulated. The up-regulation of redox enzymes might help larvae neutralize toxic reactive oxygen species produced by host plants as defense [33]. The promotion of cell division-based larval growth during the 1–3 larval period came from the following observations. First, up-regulated genes outnumbered down-regulated genes in the category ‘structure and adhesion’, indicating that more cytoskeleton components, adhesion molecules, cuticle proteins, and other structural proteins were produced. Second, more than 77% of ‘DNA replication’ genes were up-regulated, compared with less than 6% of down-regulated ‘DNA replication’ genes, indicating that cell division activity was enhanced during this larval period.

During the 3–5 day larval period, up-regulated genes encoding cellular structural components and adhesion molecules continued to significantly outnumber down-regulated genes, indicating that more structural proteins and adhesion molecules were produced to promote larval growth and development. However, more genes involved in cell division including cell cycle regulators, helicases/DNA repair, and DNA replication were down-regulated than up-regulated, indicating that cell division activity was down-regulated during the 3–5 day larval period. On the other hand, larval size continues to grow during this period [41], suggesting that larval growth might be mainly through the expansion of cell sizes. Other main differences in comparison with the 1–3 day larval period were that down-regulated genes were about equal or slightly outnumbered up-regulated genes in the category ‘RNA metabolism’, indicating that RNA synthesis and processing reached a plateau during this larval stage. Also for the genes in the category ‘protein metabolism’, instead of up-regulation of more genes involved in protein synthesis as observed in the 1–3 day larval period, up-regulated genes in the 3–5 day larval stage are mainly involved in protein modification and degradation. These results suggest that the rate of protein synthesis reached a plateau in the 3–5 day larval stage as well, whereas protein modification such as glycosilation and methylation was enhanced, which is consistent with the postulation that larval growth was achieved through cell expansion and differentiation rather than cell division. Many proteases including trypsins, chymtrypsins, cysteine proteases, and various carboxypeptidases play a role in food digestion in the midgut [42–45]. There are also a large number of protease inhibitors in the midgut, possibly serving as protection to inhibit the detrimental activity of ingested host proteases during feeding [46, 47]. The increased synthesis of digestive proteases and protective protease-inhibitors is consistent with the observation that five-day old larvae ingest the largest amount of host fluid [41].

In comparison with the promotion of larval feeding and growth during the 1–3 day and 3–5 day larval periods, a shift in gene expression during the 5–7 day larval stage was to prepare for the transition from larva to the non-feeding puparium and pupa. The major characteristics for the 5–7 day larval stage were that down-regulated genes significantly outnumbered up-regulated genes in ‘adhesion molecules’ and ‘cuticle protein’. The overall down-regulation of adhesion molecules and cuticle proteins indicated that cell growth and expansion were ceased during this period under our experimental conditions, which was again consistent with phenotypic observation [41]. On the other hand, protein synthesis was enhanced during the 5–7 day larval stage based on the larger percentages of up-regulated genes in ‘ribosomal protein’, ‘protein translation’, ‘folding/chaperone’, ‘tRNA synthesis’, and ‘protein transport’. The biological significance of the overall enhancement of nutrient metabolism, protein synthesis, and transport activity observed in the 5–7 day larval stage, however, was not as apparent as in other stages. Since larval growth appeared stopped in 7-day old larvae, it was likely that the energy and intermediates produced by enhanced nutrient metabolism and increased protein synthesis were used for a broad adjustment for preparation to transit to the next puparium stage. Consistent with this notion, genes encoding vitellogenins for nutrient storage were up-regulated (S3D Table). A much greater percentage of genes involved in the synthesis of amino acids and other nitrogen-containing compounds were also up-regulated, suggesting that the larvae were preparing nutrients for entering into next developmental stage.

### Changes in gene expression and pathways during the larva/pupa transition

The most obvious change in gene expression during the transition from 7-day larvae to pupae was the greater percentage of down-regulated genes in the categories ‘nutrient metabolism’, ‘RNA metabolism’, ‘redox/detoxification’, ‘protein metabolism’, and ‘transport’. This observation is consistent with phenotypic observation that the pupal stage of insect species is a transition stage for morphogenesis with overall subdued metabolic activity. Interestingly, up-regulated genes significantly outnumbered down-regulated genes in the category ‘structure and adhesion’. Since pupae do not grow, the overall up-regulation of genes encoding structural components suggested that a process of remaking of insect structures, namely conversion of larval structures into adult structures, underwent actively during this seemingly dormant stage. This process could involve in digestion of some larval structures and production of new structures suitable for cells in adults. The possibly increased digestion activity of larval structural proteins was suggested by the larger proportion of up-regulated genes in the subcategory ‘proteasome/ubiquilation’, which contain genes involved in proteasome-mediated protein degradation. The preparation for the transition to adult stage could also be seen because up-regulated genes outnumbered down-regulated genes in the subcategories ‘neuro-transmitter’ and ‘sensory transduction’, which are known to be enhanced in adult insects [48]

### Changes in gene expression and pathways during pupa/adult transition

During the transition from pupa to adult, energy metabolism and amino acid synthesis were partially restored as seen from the fact that up-regulated genes outnumbered down-regulated genes in ‘TCA cycle/energy metabolism’ and ‘amino acid metabolism’. As expected, down-regulated genes significantly outnumbered up-regulated genes in the category ‘structure and adhesion’ since adults do not grow and eggs in adults are single cells, which may contain less of these types of structural proteins. The most remarkable change in gene expression during the pupa/adult transition was that up-regulated genes hugely outnumbered down-regulated genes in the category ‘RNA metabolism’, suggesting that transcription and RNA processing was enhanced in adults. Protein synthesis was enhanced too based on the fact that higher percentages of genes were up-regulated in the subcategories ‘ribosomal proteins’ and ‘protein folding/chaperones’ and ‘protein transport’. The biological significance of enhanced transcription and translation remains to be determined. Hessian fly adults do not feed and do not grow. Eggs in Hessian fly are produced during the late pupal stage. Therefore, the enhanced transcription and translation were unlikely linked with adult growth or egg production. It is interesting to note that up-regulated genes outnumbered down-regulated genes in the subcategories ‘cell cycle’, ‘chromatin’, ‘helicase/DNA repair’, and ‘DNA replication’. All these genes are involved in cell division. Since adult flies do not grow, it is likely that the enhanced activity of transcription and translation is to produce components for cell division inside eggs after they are fertilized.

In conclusion, this study systematically identified genes that are differentially expressed in different developmental stages of a gall midge insect that is an important pest of agriculture and a model system for studying plant—insect interaction. Functional identification of the differentially expressed genes provided an explanation, at the molecular level, to the physiologies observed phenotypically in different developmental stages of the insect. The availability of the comprehensive data sets of differentially expressed genes and their functions shall lay a ground for future research to either identify critical genes for practical applications or to reveal biochemical regulatory mechanisms. The dataset shall also be very useful for comparative research with other insect species.

## Materials and Methods

### Insect and sample collection

Hessian flies used in this study were biotype GP, derived from a colony collected in Scott County, Kansas, in 2005 [49]. A colony has been continuously maintained in the greenhouse on the susceptible wheat cultivar ‘Karl 92’ since that initial collection.

For sample collection, 20 wheat seeds were planted in 10-cm-diameter pots filled with PRO-MIX ‘BX’ potting mix (Hummert Inc., Earth City, MO) in a growth chamber programmed at 20:18°C (L:D) with a photoperiod of 14:10 (L:D) h. When wheat seedlings reached the 1.5 leaf stage (stage 11 on Zadoks scale), the plants were infested with 0.5 Hessian fly females per plant, on average, by confining the insects in a screened cage. It usually takes 4–5 days for eggs to hatch under this condition. The exact time for larvae to reach the feeding site was determined by dissecting plants to examine if larvae had reached the feeding site at the expected time period. When the first larva was found at the feeding site, that time was set at zero and larval age started counting from that time.

Larvae were collected at day 1, 3, 5, and 7, respectively, by dissecting plants to expose the insects. The dissected plants were soaked in a micro-centrifuge tube that contained water. Hessian fly larvae fell into the water. After enough insects were collected in the tube, water was removed and insects were frozen in liquid nitrogen for RNA extraction. Pupae were collected in the same way at approximately day 12, when body fluid of insects turned from white to red. Adult females were collected randomly from a flat, and these females were presumably mated since adult flies mate right after emergence.

Three independent biological replicates for each stage of insects were collected and analyzed.

### Total RNA extraction and quantification

Total RNA was extracted using TRI reagent (Molecular Research Center Inc, Cincinnati, OH, U.S.A.), following the protocol provided by the manufacturer. RNA concentration was determined using a Nanodrop *ND-2000* spectrophotometer (NanoDrop Technologies Inc., Wilmington, DE). Quality of the RNA samples was determined using an Agilent TapeStation Bioanalzer (Agilent Technologies, Palo Alto, CA).

### RNA library construction and sequencing

RNA libraries were generated according to Illumina’s sample preparation instructions (Illumina, San Diego, CA). Briefly, approximately 20 μg of total RNA from each sample was digested with DNase I (Sigma, St. Louis) to remove potential DNA contamination. mRNA was then purified by oligo(dT) magnetic beads and fragmented into 100–400 bp fragments. cDNA was produced from the RNA fragments using reverse transcriptase (Invitrogen, Carlsbad) with random hexamers as primers. An Agilent TapeStation Bioanalzer (Agilent Technologies, Palo Alto, CA) was used to qualify and quantify the libraries. Libraries were sequenced using an Illumina HiSeq2000 system (Illumina Inc. San Diego, CA).

### Analysis of RNA-Seq data

Raw RNA-Seq reads were subjected to adaptor and quality trimming using Trimmomatic (version 0.32) [35] and the resulting clean reads were aligned to the Hessian fly draft genome sequence (http://agripestbase.org/hessianfly/) using Genomic Short-read Nucleotide Alignment Program (GSNAP) [37]. The uniquely aligned reads were used to determine the read depth per annotated gene in each sample by an in-house Perl script. To test the null hypothesis that no difference in gene expression existed of each gene between two groups, the generalized linear model method, assuming negative binomial distribution of read counts implemented in the DESeq2 package (version 1.4.5), was used to compute a p-value for each gene [38]. The parameter of “Independentfiltering = yes” in DESeq2 was setup to filter genes that were unlikely to be differentially expressed. The genes survived from the filtering are called informative genes. A FDR (false discovery rate) approach was used to convert p-values to q-values to account for multiple tests [50]. Genes with q-values no larger than 5% were declared to be differentially expressed.

### BLASTX to annotate transcripts

Sequences of a set of Hessian fly transcripts (N = 18,832) were used to search homologous hits in the GenBank non-redundant protein squence database (nr) using BLASTX. For each transcript, only the best hit with the E-value no larger than 1e-30 was reported.

### Classification of genes according to their functions

Based on the Genbank search results, the gene-models with known functions were divided into eight different functional categories according to their GO terms (http://www.uniprot.org/uniprot/) [39]. The eight categories are ‘nutrient metabolism’, ‘reduction/oxidation (redox) and detoxification’, ‘structure and adhesion’, ‘RNA metabolism’, ‘protein metabolism’, ‘transport’, ‘regulatory proteins’ and ‘SSGPs’. Each category was further divided into sub-categories, again based on their GO terms. The subcategories were described in the results section.

### Accession no for RNAseq data

SAMN04943327, SAMN04943328, SAMN04943329, SAMN04943330, SAMN04943331, SAMN04943332, SAMN04943333, SAMN04943334, SAMN04943335, and SAMN04943336 (BioProject ID PRJNA320634).

## Supporting Information

S1 Fig Percentages of total up- (red bars) and down-regulated (blue bars) genes between samples from two successive stages of Hessian fly. ‘3 vs 1’, ‘5 vs 3’, ‘7 vs5’, ‘P vs 7’, and ‘A vs P’ represent comparisons made between 3- versus 1-day larvae, 5- versus 3-day larvae, 7- versus 5-day larvae, pupae versus 7-day larvae, and adults versus pupae.
(DOC)

S2 Fig Volcano plots of RNA-Seq comparisons. The volcano plot compares gene expression between two neighboring stages. Negative log10 p-values (y-axis) from differential expression tests were plotted versus the log2 fold change for each gene. Each dot represents a gene. The horizontal dash line indicates the significant cutoff that was used to declare significantly differential expression. Blue and red highlight up- and down-regulations, respectively.
(PDF)

S1 Table Total gene models that were found to have the corresponding transcripts detected in at least one of the RNAseq samples. Gene identification number (GeneID), Chromosome (Chr), strand orientation (Ori), exon starting (Start) and ending (End) sites, Exon size, and transcrpt sequences were obtained from the Hessian fly genome database (http://agripestbase.org/hessianfly/).
(XLS)

S2 Table Comparison of transcript abundance based on RNAseq data between two samples from successive developmental stages of Hessian fly. Three biological replicates (rep) were conducted for each sample. RPKM represents Reads Per Kilobase per Million mapped reads. S2 contains five separate sub-Tables, from S2A to S2E as shown below. S2A: Comparison between 3-day versus 1-day larvae. S2B: Comparison between 5-day versus 3-day larvae. S2C: Comparison between 7-day versus 5-day larvae. S2D: Comparison between Pupae versus 7-day larvae. S2E: Comparison between adults versus pupae.
(XLSX)

S3 Table Classification of informative gene models of Hessian fly into different categories and subcategories. This table contains 11 sub-tables, each of which is shown in a different excel sheet in the same excel file. The sub-tables are as following: S3A: Classification of total informative gene models of Hessian fly into different categories and subcategories. S3B 3-1U: Classification of up-regulated genes when the samples from 3-day larvae compared with the samples from 1-day larvae. S3B 3-1D: Classification of down-regulated genes when the samples from 3-day larvae compared with the samples from 1-day larvae. S3C 5-3U: Classification of up-regulated genes when the samples from 5-day larvae compared with the samples from 3-day larvae. S3C 5-3D: Classification of down-regulated genes when the samples from 5-day larvae compared with the samples from 3-day larvae. S3D 7-5U: Classification of up-regulated genes when the samples from 7-day larvae compared with the samples from 5-day larvae. S3D 7-5D: Classification of down-regulated genes when the samples from 7-day larvae compared with the samples from 5-day larvae. S3E P-7U: Classification of up-regulated genes when the samples from pupae compared with the samples from 7-day larvae. S3E P-7D: Classification of down-regulated genes when the samples from pupae compared with the samples from 7-day larvae. S3F A-PU: Classification of up-regulated genes when the samples from adults compared with the samples from pupae. S3F A-PD: Classification of down-regulated genes when the samples from adults compared with the samples from pupae.
(XLSX)

## References

[1] Gagné RJ. A catalog of the Cecidomyiidae (Diptera) of the world. Entomol Soc Washington 2004; 25.

[2] Skuhravá M, Skuhravý V. Species richness of gall midges (Diptera: Cecidomyiidae) in Europe (West Palaearctic): biogeography and coevolution with host plants. *Acta Soc Zool Bohem*. 2009; 73: 87–156.

[3] Harris MO, Stuart JJ, Mohan M, Nair S, Lamb RJ, Rohfritsch O. Grasses and gall midges: plant defense and insect adaptation. *Annu Rev Entomol*. 2008; 48: 549–577. 10.1146/annurev.ento.48.091801.112559 12460937

[4] Bentur JS, Cheralu C, Rao PRM. Monitoring virulence in Asian rice gall midge populations in India. *Entomol Exp Appl*. 2008; 129: 96–106.

[5] Gould F. Sustainability of transgenic insecticidal cultivars: Integrating pest genetics and ecology. *Annu Rev Entomol*. 1998; 43: 701–726. 15012402 10.1146/annurev.ento.43.1.701

[6] Sánchez L. Sex-determining mechanisms in insects based on imprinting and elimination of chromosomes. *Sex Dev*. 2014; 8: 83–103. 10.1159/000356709 24296911

[7] Stuart JJ, Chen MS, Harris MO. Hessian fly *Genome Mapping and Genomics in Animals*. *Volume 1*: *Genome Mapping and Genomics in Arthropods* (eds. Hunter W. & Kole C.), 2008; pp. 93–100. Springer Verlag, Berlin.

[8] Stuart JJ, Chen MS, Shukle R, Harris MO. Gall midges (Hessian flies) as plant pathogens. *Annu Rev Phytopath*. 2012; 50: 339–358. 10.1146/annurev-phyto-072910-095255 22656645

[9] Chavalle S, Censier F, San G, Gomerz MY, De Proft M. Protection of winter wheat against orange wheat blossom midge, *Sitodiplosis mosellana* (Géhin) (Deiptera: Cecidomyiidae): efficacy of insecticides and cultivar resistance. *Pest Manag Sci*. 2015; 71: 783–790. 10.1002/ps.3855 25044220

[10] Mezghani-Khenakhem M, Bouktila D, Casse N, Maaroufi H, Makni M, Makni H. Development of new polymorphic microsatellite loci for the barley stem gall midge, *Mayetiola hordei* (Diptera: Cecidomyiidae) from an enriched library. *Int J Mol Sci*. 2012; 13: 14446–14450. 10.3390/ijms131114446 23203074 PMC3509590

[11] Censier F, Chavalle S, De Proft M, Bodson B. Study on the sensitivity of three oat varieties to the saddle gall midge, *Haplodiplosis marginata* (von Roser) (Diptera: cecidomyiidae). *Commun Agric Appl Biol Sci*. 2013; 78: 287–292. 25145247

[12] Roubos CR, Liburd OE. Pupation and emergence of blueberry gall midge, Dasineura oxycoccana (Diptera: Cecidomyiidae), under varying temperature conditions. *Florida Entomol*. 2010; 93: 283–290.

[13] DeAngelis J. Biology and control of honeylocust pod gall midge. *Ornament Northwest Arch*. 1989; 13: 15.

[14] Hernandez LM, Guzman YC, Martínez-Arias A, Manzano MR, Selvaraj JJ. The bud midge *Prodiplosis longifila*: Damage characteristics, potential distribution and presence on a new crop host in Colombia. *Springerplus*. 2015; 4: 205 10.1186/s40064-015-0987-6 25977894 PMC4424221

[15] Zhao C, Escalante LN, Chen H, Benatti TR, Qu J, Chellapilla S et al A massive expansion of effector genes underlies gall-formation in the wheat pest *Mayetiola destructor*. *Curr Biol*. 2015; 25: 613–620. 10.1016/j.cub.2014.12.057 25660540

[16] Byers RA, Gallun RL. Ability of the Hessian fly to stunt winter wheat. I. Effect of larval feeding on elongation of leaves. *J Econ Entomol*. 1972; 65: 955–958.

[17] Chen MS, Liu X, Wang H, El Bouhssini M. Hessian fly (Diptera: Cecidomyiidae) interactions with barley, rice, and wheat seedlings. *J Econ Entomol*. 2009, 102; 1663–1672. 19736782 10.1603/029.102.0434

[18] Ratcliffe RH, Cambron SE, Flanders KL, Bosque-Perez NA, Clement SL, Ohm HW. Biotype composition of Hessian fly (Deptera: Cecidomyiidae) populations from the Southeastern, Midwestern, and Northwestern United States and virulence to resistance genes in wheat. *J Econ Entomol*. 2000; 93: 1319–1328. 10985049 10.1603/0022-0493-93.4.1319

[19] Garcés-Carrera S, Knutson A, Wang H, Giles KL, Huang F, Whitworth RJ et al Virulence and biotype analysis of Hessian fly (Diptera: Cecidomyiidae) population from Texas, Louisiana, and Oklahoma. *J Econ Entomol*. 2014; 107(1): 417–423. 24665728 10.1603/ec13372

[20] Chen MS, Liu X, Yang Z, Zhao H, Shukle R, Stuart JJ et al Unusual conservation among genes encoding small secreted salivary gland proteins from a gall midge. *BMC Evol Biol*. 2010; 10: 296 10.1186/1471-2148-10-296 20920202 PMC2955719

[21] Khajuria C, Williams CE, El Bouhssini M, Whitworth RJ, Richards S, Stuart JJ et al Deep sequencing and genome-wide analysis reveals the expansion of microRNA genes in the gall midge *Mayetiola destructor*. *BMC Genomics*. 2013; 14: 187 10.1186/1471-2164-14-187 23496979 PMC3608969

[22] Buntin GD. Hessian fly (Diptera: Cecidomyiidae) injury and loss of winter wheat grain yield and quality. *J Econ Entomol*. 1999; 92: 1190–1197.

[23] Morin RD, Bainbridge M, Fejes A, Hirst M, Kizywinski M, Pugh TJ et al Profiling the HeLa S3 transcriptome using randomly primed cDNA and massively parallel short-read sequencing. *Bio Techquiques*. 2008; 45: 81–94. 10.2144/000112900 18611170

[24] Chu Y, Corey DR. RNA sequencing: platform selection, experimental design, and data interpretation. *Nucl Acid Ther*. 2012; 22: 271–274. 10.1089/nat.2012.0367 PMC3426205 22830413

[25] Vraveley BR, Brooks AN, Carlson JW, Duff MO, Landolin JM, Yang L et al The developmental transcriptome of Drosophila melanogaster. *Nature*. 2011; 471: 473–479. 10.1038/nature09715 21179090 PMC3075879

[26] Poelchau MF, Reynolds JA, Elsik CG, Denlinger DL, Armbruster PA. RNA-seq reveals early distinctions and late convergence of gene expression between diapauses and quiescence in the Asian tiger mosquito, *Aedes albopictus*. *J Exp Biol*. 2013; 216: 4082–4090. 10.1242/jeb.089508 23913949 PMC3797908

[27] Poelchau MF, Reynolds JA, Elsik CG, Denlinger DL, Armbruster PA. Deep sequencing reveals complex mechanisms of diapauses preparation in the invasive mosquito, *Aedes albopictus*. *Proc R Soc B*. 2013; 280: 20130143 10.1098/rspb.2013.0143 23516243 PMC3619507

[28] Akbari OS, Antoshechkin I, Amrhein H, Williams B, Diloreto R, Sandler J et al The developmental transcriptome of the mosquito Aedes aegypti, an invasive species and major arbovirus vector. *G3 Genes/genomes/Genetics*. 2013; 3: 1493–1509. 10.1534/g3.113.006742 PMC3755910 23833213

[29] Tarrant AM, Baumgartner MF, Hansen BH, Altin D, Nordtug T, Olsen AJ. Transcriptional profiling of reproductive development, lipid storage and molting throughout the last juvenile stage of the marine copepod *Calanus finmarchicus*. *Frontiers Zool*. 2014; 11: 91. 10.1186/s12983-014-0091-8 PMC4285635 25568661

[30] Tian C, Tay WT, Feng H, Wang Y, Hu Y, Li G. Characterization of Adelphocoris suturalis (Hemiptera: Miridae) transcriptome from different developmental stages. *Sci Rep*. 2015; 5: 11042 10.1038/srep11042 26047353 PMC4457133

[31] Allen ML. Characterization of adult transcriptomes from the Omnivorous lady beetle Coleomegilla maculate fed pollen or insect egg diet. *J Genomics*. 2015; 3: 20–28. 10.7150/jgen.10385 25628762 PMC4303598

[32] Vogel H, Badapanda C, Knorr E, Vilcinskas A. RNA-sequencing analysis reveals abundant developmental stage-specific and immunity-related genes in the pollen beetle *Meligethes aeneus*. *Insect Mol Biol*. 2014; 23: 98–112. 10.1111/imb.12067 24252113

[33] Liu X, Williams CE, Nemacheck JA, Wang H, Subramanyam S, Zheng C et al Reactive oxygen species involved in plant defense against a gall midge. *Plant Physiol*. 2010; 152: 985–999. 10.1104/pp.109.150656 19965963 PMC2815885

[34] Aggawal R, Subramanyam S, Zhao C, Chen MS, Harris MO, Stuart JJ. Avirulence effector discovery in a plant galling and plant parasitic arthropod, the Hessian fly (*Mayetiola destructor*). *PloS ONE*. 2014; 9(6): e100958 10.1371/journal.pone.0100958 24964065 PMC4071006

[35] Zhao C, Shukle R, Navarro-Escalante L, Chen MS, Richards S, Stuart JJ. Avirulence gene mapping in the Hessian fly (Mayetiola destructor) reveals a protein phosphatase 2C effector gene family. *J Insect Physiol*. 2016; 84: 22–31. 10.1016/j.jinsphys.2015.10.001 26439791

[36] Bolger AM, Lohse M, Usadel B. Trimmomatic: a flexible trimmer for Illumina sequence data. *Bioinformatics*. 2014; 30: 2114–2120. 10.1093/bioinformatics/btu170 24695404 PMC4103590

[37] Wu TD, Nacu S. Fast and SNP-tolerant detection of complex variants and splicing in short reads. *Bioinformatics*. 2010; 26: 873–881. 10.1093/bioinformatics/btq057 20147302 PMC2844994

[38] Love MI, Huber W, Anders S. Moderated estimation of fold change and dispersion for RNA-seq data with DESeq2. *Genome Biol*. 2014; 15: 550 25516281 10.1186/s13059-014-0550-8 PMC4302049

[39] Magrane M, The UniProt consortium. UniProt Knowledgebase: a hub of integrated protein data. *Database*. 2011; 2011: bar009 10.1093/database/bar009 21447597 PMC3070428

[40] The Gene Ontology Consortium. Gene ontology: tool for the unification of biology. *Nat Genet*. 2000; 25(1): 25–29. 10802651 10.1038/75556 PMC3037419

[41] Gagné RJ, Hatchett JH. Instars of the Hessian fly (Diptera: Cecidomyiidae). *Ann Entomol Soc Amer*. 1989; 81: 73–79.

[42] Mittapalli O, Stuart JJ, Shukle RH. Molecular cloning and characterization of two digestive serine proteases from the Hessian fly, *Mayetiola destructor*. *Insect Mol Biol*. 2005; 14: 309–318. 15926900 10.1111/j.1365-2583.2005.00561.x

[43] Zhu YC, Liu X, Maddur AA, Oppert B, Chen MS. Cloning and characterization of chymotrypsin- and trypsin-like cDNAs from the gut of the Hessian fly [*Mayetiola destructor* (Say)]. *Insect Biochem Mol Biol*. 2005; 35: 23–32. 15607652 10.1016/j.ibmb.2004.09.006

[44] Liu X, Fellers JP, Zhu YC, Mutti NS, El Bouhssini M, Chen MS. Cloning and characterization of cDNAs encoding carboxypeptidase-like proteins from the gut of Hessian fly larvae [*Mayetiola destructor* (Say)]. *Insect Biochem Mol Biol*. 2006; 36: 665–673. 16876709 10.1016/j.ibmb.2006.05.008

[45] Chen H, Zhu YC, Whitworth RJ, Reese JC, Chen MS. Serine and cysteine protease-like genes in the genome of a gall midge and their interactions with host plant genotypes. *Insect Biochem Mol Biol*. 2013; 43: 701–711. 10.1016/j.ibmb.2013.05.006 23727407

[46] Maddur AA, Liu X, Zhu YC, Fellers JP, Oppert B, Park Y et al Cloning and characterization of protease inhibitor-like cDNAs from the Hessian fly *Mayetiola destructor* (Say). *Insect Mol Biol*. 2006; 15: 485–496. 16907835 10.1111/j.1365-2583.2006.00660.x

[47] Lopez L, Camas A, Shivaji R, Ankala A, Williams P, Luthe D. Mir-CP, a novel defense cysteine protease accumulates in maize vascular tissues in response to herbivory. *Planta*. 2007; 226: 517–527. 17351787 10.1007/s00425-007-0501-7

[48] Yamamoto D, Koganezawa M. Genes and circuits of courtship behavior in *Drosophila melanogaster*. *Nat Rev Neur*. 2013; 14: 681–692. 10.1038/nrn3567 24052176

[49] Chen MS, Echegaray E, Whitworth RJ, Wang H, Sloderbeck PE, Knutson A et al Virulence analysis of Hessian fly populations from Texas, Oklahoma, and Kansas. *J Encon Entomol*. 2009; 102: 774–780. 10.1603/029.102.0239 19449660

[50] Benjamini Y, Hochberg Y. Controlling the false discovery rate: a practical and powerful approach to multiple testing. *J Roy Statistical Society*. 1995; Series B 57: 289–300.

