# Supplementary material for: Massive Shift in Gene Expression during Transitions between Developmental Stages of the Gall Midge, Mayetiola Destructor
Source: PLoS One. 2016 May 25;11(5):e0155616. doi: 10.1371/journal.pone.0155616 (PMC4880318; doi:10.1371/journal.pone.0155616)
Supplement: S1 Fig — ‘3 vs 1’, ‘5 vs 3’, ‘7 vs5’, ‘P vs 7’, and ‘A vs P’ represent comparisons made between 3- versus 1-day larvae, 5- versus 3-day larvae, 7- versus 5-day larvae, pupae versus 7-day larvae, and adults versus pupae. (DOC) [file pone.0155616.s001.doc]

Figure S1. Percentages of total up- (red bars) and down-regulated (blue bars) genes between samples from two successive stages of Hessian fly. ‘3 vs 1’, ‘5 vs 3’, ‘7 vs5’, ‘P vs 7’, and ‘A vs P’ represent comparisons made between 3- versus 1-day larvae, 5- versus 3-day larvae, 7- versus 5-day larvae, pupae versus 7-day larvae, and adults versus pupae.
