# Supplementary material for: Massive Shift in Gene Expression during Transitions between Developmental Stages of the Gall Midge, Mayetiola Destructor
Source: PLoS One. 2016 May 25;11(5):e0155616. doi: 10.1371/journal.pone.0155616 (PMC4880318; doi:10.1371/journal.pone.0155616)
Supplement: S2 Fig — The volcano plot compares gene expression between two neighboring stages. Negative log10 p-values (y-axis) from differential expression tests were plotted versus the log2 fold change for each gene. Each dot represents a gene. The horizontal dash line indicates the significant cutoff that was used to declare significantly differential expression. Blue and red highlight up- and down-regulations, respectively. (PDF) [file pone.0155616.s002.pdf]

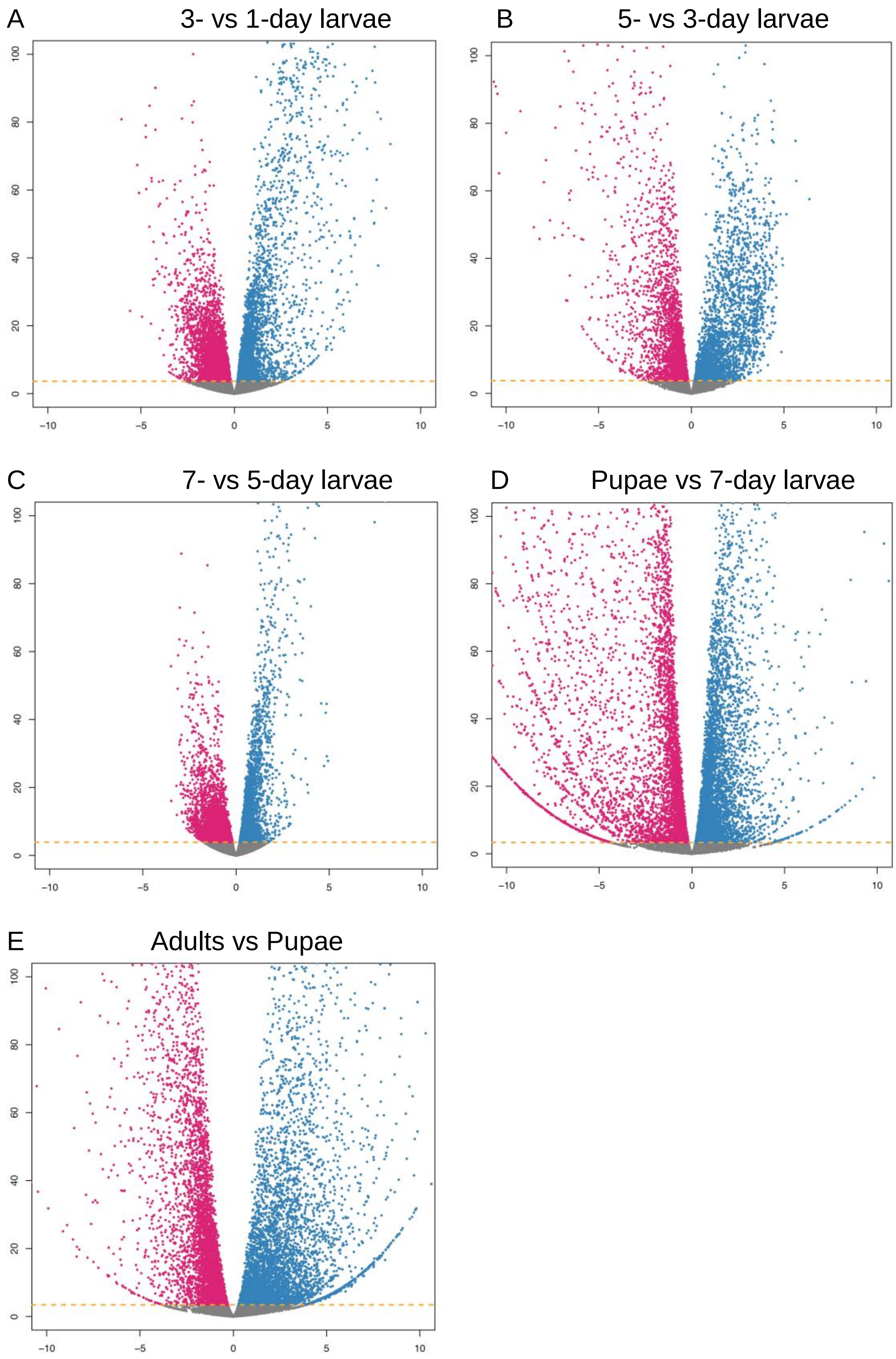
A
3- vs 1-day larvae
B
5- vs 3-day larvae
C
7- vs 5-day larvae
D
Pupae vs 7-day larvae
E
Adults vs Pupae

Figure S2. Volcano plots of RNA-Seq comparisons

The volcano plot compares gene expression between two neighboring stages. Negative log10 p-values (y-axis) from differential expression tests were plotted versus the log2 fold change for each gene. Each dot represents a gene. The horizontal dash line indicates the significant cutoff that was used to declare significantly differential expression. Blue and red highlight up- and down-regulations, respectively.
